# Case studies in applied epidemiology

**DOI:** 10.11604/pamj.supp.2017.27.1.12886

**Published:** 2017-05-28

**Authors:** Richard C Dicker

**Affiliations:** 1 Workforce and Institute Development Branch, Division of Global Health Protection, Center for Global Health, U.S. Centers for Disease Control and Prevention, Atlanta, Georgia, US

**Keywords:** Case study, applied epidemiology, field epidemiology

## Foreword

A hallmark of field epidemiology training is its focus on acquisition of practical epidemiologic knowledge and skills to address priority public health issues. The training must prepare the trainee to conduct the core functions of a field epidemiologist – investigate outbreaks, conduct public health surveillance, collect and analyze data, use epidemiologic judgment, and communicate effectively. While these functions or competencies are best learned through practice in the field under the guidance of experienced mentors, even the classroom component that usually precedes the fieldwork can help prepare the trainee. For example, to supplement a lecture on the steps of an outbreak investigation, the unfolding circumstances of an actual outbreak can be presented in the classroom, and trainees could be asked what decisions they would make, what hypotheses they would consider, what statistics they might calculate (and given the data, calculate them), what conclusions they might draw from the data, and so on.

The first outbreak known to be used in this way to teach epidemiologic field investigation principles and methods is the now legendary outbreak of gastroenteritis following a church supper in Oswego, New York in 1940. The Oswego Problem was used as a teaching example at the nearby Albany Medical College in 1942. Alexander Langmuir brought Oswego to the Communicable Disease Center (CDC, now the Centers for Disease Control and Prevention), where he used it to teach outbreak investigation to the first cohort of Epidemic Intelligence Service (EIS) Officers in 1951 [1], Oswego was soon followed by Epidemic Disease in South Carolina and many others.

For many years these teaching examples were called "problems" or "exercises", but neither term seemed entirely satisfactory as a descriptor. In 1988, EIS training staff looked for a new name, and settled on “case study.” However, these applied epidemiology cases studies differ in a number of ways from what are called case studies in other disciplines, particularly the case study based on a single patient in clinical medicine or psychology or the case studies used in business schools. Business-school case studies are in-depth stories, ranging from a few pages to over a hundred pages in length, that present issues for which one or more decisions are needed, often without a right answer; a limited number of questions are posed at the end [2]. Students read the case study as homework, and come to class prepared to discuss the questions. In contrast, applied epidemiology case studies are usually read by the trainees in class, often out loud, stopping to answer questions that are interspersed throughout, without looking ahead. The questions can ask for a decision, but often they instruct the trainees to perform calculations, draw graphs, generate lists, interpret data, or consider the pros and cons of different approaches.

Applied epidemiology case studies quickly became a cornerstone of the classroom modules of the EIS Program, and later of Field Epidemiology Training Programs (FETPs) around the world. One reason is that they are consistent with the principles of adult learning. They are job-relevant, focusing on tasks, skills, and knowledge of surveillance, outbreak investigation, and data analysis / interpretation that trainees will use during their field placements. They use actual examples to reinforce concepts. They require active participation and problem solving. They are used in a collaborative environment in which trainees contribute to the learning based on their own experience and insights, so trainees learn from their peers as well as from the instructors. In addition, they are fun, consistently receiving the highest ratings on student evaluations of any component of classroom training.

Trainees always prefer that teaching materials use local examples to which they can readily relate. But writing a new case study requires considerable time, effort, and access to the original data, and few FETPs have taken the initiative. Consequently, until recently, most case studies used by FETPs were borrowed from the EIS program, and were U.S.-based.

To address the dearth of locally developed case studies, particularly in Africa, the Africa Field Epidemiology Network (AFENET) and Emory University’s Rollins School of Public Health designed and conducted a workshop to guide African FETP staff or partners in the development of local case studies. The case studies included in this supplement are the products of that workshop. We are pleased to add these case studies to the library of training materials available to FETP trainers, university faculty, and others who wish to teach field epidemiology in an engaging and interactive way.
